# Age-related deficits in synaptic plasticity rescued by activating PKA or PKC in sensory neurons of *Aplysia californica*

**DOI:** 10.3389/fnagi.2015.00173

**Published:** 2015-09-03

**Authors:** Andrew T. Kempsell, Lynne A. Fieber

**Affiliations:** Department of Marine Biology and Ecology, Rosenstiel School of Marine and Atmospheric Science, University of MiamiMiami, FL, USA

**Keywords:** short term memory, long term potentiation, pleural ganglion, pedal ganglion, marine invertebrate

## Abstract

Brain aging is associated with declines in synaptic function that contribute to memory loss, including reduced postsynaptic response to neurotransmitters and decreased neuronal excitability. To understand how aging affects memory in a simple neural circuit, we studied neuronal proxies of memory for sensitization in mature vs. advanced age *Aplysia californica* (Aplysia). L-Glutamate- (L-Glu-) evoked excitatory currents were facilitated by the neuromodulator serotonin (5-HT) in sensory neurons (SN) isolated from mature but not aged animals. Activation of protein kinase A (PKA) and protein kinase C (PKC) signaling rescued facilitation of L-Glu currents in aged SN. Similarly, PKA and PKC activators restored increased excitability in aged tail SN. These results suggest that altered synaptic plasticity during aging involves defects in second messenger systems.

## Introduction

Aging affects several cellular properties of neurons, causing a progressive decline in neuronal excitability and synaptic function, and culminates in sensory and motor deficits and memory loss (Kumar and Foster, 2007; Kumar et al., 2012). Decreased synaptic strength during aging is the result of declines in postsynaptic responsiveness including decreases in level of expression of ionotrophic glutamate receptors (iGluR; Magnusson et al., 2002). We previously demonstrated that L-glutamate (L-Glu) activated excitatory currents in isolated tail sensory neurons (SN) of the marine snail *Aplysia californica* (Aplysia; Carlson and Fieber, 2011; Carlson et al., 2012), but that L-Glu-induced current amplitude was decreased significantly in old tail SN (Fieber et al., 2010; Kempsell and Fieber, 2014), suggesting that losses in SN excitability are a hallmark of aging.

Direct stimulation of the tail initiates the tail withdrawal reflex (TWR). TWR involves identified SN in the pleural ganglia and motoneurons (MN) in the pedal ganglia (Walters et al., 1983a, 2004). Distinct forms of nonassociative learning can be evoked in TWR. Sensitization in TWR is an increase in reflexive response following an aversive shock to part of the body. Short- and long-term forms of sensitization can be induced depending on the quantity and pattern of training (Walters et al., 1983b; Stopfer and Carew, 1996; Stopfer et al., 1996; Sutton et al., 2002; Glanzman, 2009). These changes in behavior are reflected in concurrent changes in the efficacy between tail SN and MN synapses.

The molecular pathways involved in these forms of learning are well-studied (Kandel, 2001; Glanzman, 2008). For example, the neuromodulator serotonin (5-HT) is released at the SN-MN synapse during sensitization, resulting in increased tail SN excitability and an increase in tail SN-MN transmission (Brunelli et al., 1976; Walters et al., 1983b; Glanzman et al., 1989; Mackey et al., 1989; Mercer et al., 1991; Levenson et al., 1999; Marinesco and Carew, 2002; Philips et al., 2011). 5-HT released during synaptic facilitation activates protein kinase A (PKA)- and protein kinase C (PKC)-induced signaling in both the SN and MN (Glanzman, 2008; Villareal et al., 2009). Activation of these second messenger cascades results in dynamic processes involved in the formation and maintenance of memory. For example, in short-term facilitation (≤30 min), a brief pulse of 5-HT activates adenylyl cyclase in presynaptic neurons, causing an increase in cyclic adenosine monophosphate (cAMP), activation of PKA, and closing of K^+^ channels. Closure of K^+^ channels increases action potential (AP) duration and thus raises presynaptic cytosolic Ca^2+^, causing increased release of neurotransmitter into the synaptic cleft and temporary strengthening of the synapse. Longer exposures to 5-HT cause the prolonged activation of presynaptic PKA and induce long-term facilitation (≥24 h). Long-term facilitation requires both transcription and translation. Prolonged PKA activity results in the phosphorylation of transcription factors such as CREB-1, stimulating RNA and protein synthesis (Van der Zee et al., 1997, 2004; Oh et al., 2009).

Our prior studies describing aging in Aplysia TWR suggested that changes in tail SN excitability, whether in isolated SN or in SN of reduced ganglia-tail preparations, are reliable and electrophysiologically tractable features of the aging of TWR. In this study we investigated proxies for synaptic plasticity in tail SN during aging, including 5-HT-induced changes in excitability. We found that aging impairs the normal 5-HT-mediated response of enhancing SN excitability. Activating PKA and PKC signaling in aged SN partially restored 5-HT-induced excitability.

## Materials and Methods

Full or half sibling cohorts of Aplysia from the University of Miami National Resource for Aplysia were raised from egg masses of wild-caught animals. They were reared on an ad-libitum diet consisting of *Gracilaria ferox* and *Agardhiella* *subulata* as described previously (Gerdes and Fieber, 2006). Sexual maturity for a cohort of animals was designated as the day the first egg mass was laid.

Electrophysiological experiments were executed at maturity and when the animals reached stage aged II, as described previously (Kempsell and Fieber, 2014). Mature animals were age 7–8 months and sexually reproductive for <1 month at the time of experiments. Aged II animals were age 12–13 months, and had reduced performance in the righting reflex, TWR, and biting response compared to mature and aged I siblings (Kempsell and Fieber, 2014).

### Whole-Cell Voltage Clamp of Isolated, Cultured SN

Pleural ventral caudal (PVC) SN were isolated from pleural ganglia, dissociated onto 35 × 10 mm polystyrene culture plates coated with poly-D-lysine, and the primary cultures stored at 17°C in a humidified atmosphere as described (Fieber, 2000; Fieber et al., 2013). Whole-cell voltage clamp recordings were made 24–48 h after plating using 1.5 mm outer diameter borosilicate glass microelectrodes and an Axopatch 200B patch clamp amplifier connected to a PC equipped with a Digidata 1440 A/D converter and pClamp10 software (Molecular Devices, Sunnyvale, CA, USA). Experiments were performed in continuously flowing artificial seawater (ASW) while L-Glu was applied at 1 mM in ASW for 100 ms via a micropipette attached to a picospritzer positioned ~30 μm from the cell body at an angle of ~45° with the perfusion outflow pipette. SN responses to L-Glu were recorded from a holding potential of −70 mV every 60 s. Five pre-treatment L-Glu-evoked currents were recorded and the baseline response was calculated from their average. 5-HT (20 μM), dibutyryl adenosine 3′,5′-cyclic monophosphate (dbcAMP; 20 μM), or phorbol 12-myristate 13-acetate (PMA; 50 nM) in ASW was then perfused into the recording chamber for 10 min while L-Glu-evoked currents were recorded. After 10 min treatment, washout of treatment began, and L-Glu-evoked currents continued to be recorded every 60 s for an additional 10 min (10 min washout). PVC SN from the same cohort of animals were studied at maturity and aged II.

### Intracellular Recording of Tail SN in Semi-Intact Tail Preparation

Electrophysiological experiments in semi-intact preparations involved tail SN responses to injected current before and after application of PMA or dbcAMP from the same cohort of animals at maturity and aged II. Animals were anesthetized by injection of isotonic MgCl_2_ (~50% animal weight by volume) into the body cavity. A ganglia-tail preparation was made, consisting of either the left or right pleural-pedal hemiganglia that remained connected to the tail by nerve p9 (Kempsell and Fieber, 2015); all other connectives were severed. The ganglia-tail preparation was pinned tightly to a Sylgarded dish. Use of a semi-intact tail preparation allowed for verification of tail SN response to tail touch. The other ganglia were removed from the remaining animal tissue to euthanize it, and unneeded ganglia and tissue were discarded. Ganglia were surgically desheathed and maintained in ASW via a gravity-fed perfusion pipette located ~5 mm from the ganglia. Tail SN had resting potentials of −40–55 mV and were not spontaneously active, but when receptive fields on the tail were stimulated, produced AP of 60–100 mV amplitude (Walters et al., 2004).

Three pre-treatment (-PMA or -dbcAMP) tests were recorded with a 5 min interval between tests, and the baseline response illustrated in the figures was calculated from their average. Post-treatment test responses were then evoked at 5, 15, and 30 min after PMA or dbcAMP application for 10 min from the perfusion pipette. To measure excitability in tail PVC SNs, a protocol was modified from previous experiments (Fieber et al., 2010; Kempsell and Fieber, 2014). Depolarizing current was applied to SN in increasing increments of 0.1 nA for 500 ms until the SN fired a single AP, then the single AP evoked by this depolarization was confirmed 3 times (baseline response; 5 min interval between tests). PMA (50 nM) or dbcAMP (20 μM) was perfused onto the pleural-pedal ganglion for 10 min at concentrations known to induce forms of modulation including synaptic facilitation, increased AP duration and increased excitability in tail SNs (Carlson and Fieber, 2011; Carlson et al., 2012). After treatments of either PMA or dbcAMP, the same depolarizing current used to establish the baseline was applied, and the number of AP produced in the SN was counted.

Glass capillary microelectrodes of 5–15 MΩ resistance were used for intracellular recordings in tail SN. All recordings were made at room temperature of ~23°C using pClamp10 software with BRAMP-01R and ELC-01MX amplifiers (ALA Scientific Instruments, Farmingdale, NY, USA) connected to a PC and Digidata 1440A A/D converter.

### Solutions

Extracellular solution consisted of ASW containing (mM) 417 NaCl, 10 KCl, 10 CaCl_2_, 55 MgCl_2_, and 15 HEPES-NaOH, pH 7.6. The pipette solution for intracellular recordings in intact ganglia consisted of 3 M KCl. Intracellular solution for whole-cell voltage clamp recordings consisted of (mM) 458 KCl, 2.9 CaCl_2_ (2 H_2_O), 2.5 MgCl_2_^.^(6 H_2_O), 5 Na_2_ATP, 1 EGTA, and 40 HEPES-KOH, pH 7.4. Solutions containing L-Glu, 5-HT-HCl, PMA, or dbcAMP sodium salt in ASW were prepared daily from 0.5 M stocks as previously reported (Carlson et al., 2012). All reagents were from Sigma-Aldrich (St. Louis, MO, USA).

### Data Acquisition and Analysis

Data were expressed as mean ± SE. Significant differences, such as that occurring during 5-HT treatment, were determined by one-way within subjects (repeated-measures) ANOVA. After the ANOVA, individual differences were compared against baseline using Tukey’s *post hoc* test. All analyses were performed using the open source R statistical program (Vienna, Austria). Differences at *p* ≤ 0.05 were accepted as significant.

## Results

Sexual maturity occurred by age 7 months and median lifespans were 362 and 369 days for the two cohorts studied. Morphological and aging characteristics in aging Aplysia were reported in a previous study (Kempsell and Fieber, 2014).

### 5-HT Facilitated Mature but not Aged II Tail SN Response to L-Glu

**Figure 1:**
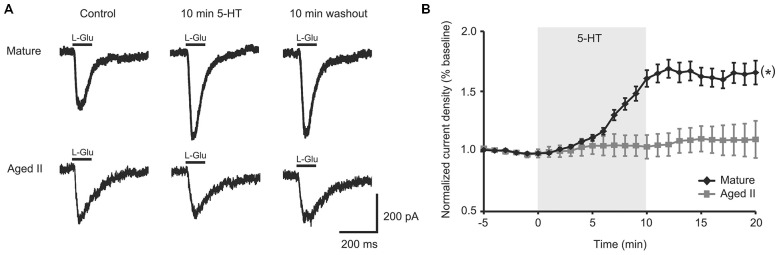
**5-HT-induced facilitation of L-glutamate (L-Glu) currents was present in mature tail sensory neurons (SN), but absent in aged II tail SN. (A)** Representative currents evoked in response to 100 ms pulse of L-Glu in isolated SN before 5-HT, after 10 min 5-HT, and after 10 min washout of 5-HT. **(B)** While current density increased significantly following 5-HT treatment in isolated SN from mature animals, no change was observed in aged II SN. (*) denotes significant overall increase in current density at *p* ≤ 0.05 via repeated measures ANOVA (*n* = 16 for mature, *n* = 20 for aged II). *Post hoc* comparisons responsible for this difference are summarized in results.

A 100 ms pulse of L-Glu (1 mM) evoked excitatory currents in SN from mature Aplysia and in SN from their aged II siblings (Figure 1A), although L-Glu current density was significantly smaller in aged II SN (mature: 2.5 ± 0.6 pA/pF, aged II: 1.3 ± 0.2 pA/pF; *p* ≤ 0.05, 2-sample *t*-test), consistent with previously reported findings (Fieber et al., 2010). 5-HT treatment significantly potentiated L-Glu current density at all time points after treatment was initiated in mature SN but not in SN from aged II sibling animals (Figure 1B; *p* ≤ 0.05, repeated measures ANOVA; mature *n* = 16 aged II *n* = 20). Facilitation of mature SN L-Glu evoked responses were maintained even following 10 min ASW washout (*p* ≤ 0.05 for 7–20 min time points compared to baseline; Tukey’s *post hoc* tests). In SN isolated from aged II animals, 5-HT treatment did not change L-Glu current density. Thus, 5-HT-induced facilitation of L-Glu-evoked responses was absent in aged II SN.

### Activators of PKA and PKC Rescued Facilitation of L-Glu Currents in Isolated Aged II SN

5-HT-induced facilitation in tail SN and MN is dependent on second messenger cascades including PKA- and PKC-mediated signaling (Kandel, 2001; Glanzman, 2008). The aging failure in 5-HT-induced facilitation led us to hypothesize that either deficient 5-HT receptors or altered G-protein-dependent cascades might be responsible. We and others previously demonstrated that PMA facilitated L-Glu currents in Aplysia SN (Carlson and Fieber, 2012) and MN (Braha et al., 1990; Villareal et al., 2009). Furthermore, cAMP was previously shown to increase tail SN excitability and facilitate tail SN-MN transmission (Brunelli et al., 1976; Baxter and Byrne, 1990).

**Figure 2:**
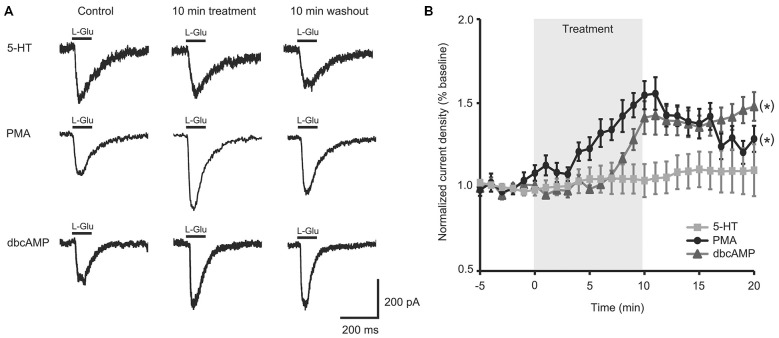
**Activators of protein kinase A (PKA) and protein kinase C (PKC) signaling rescued facilitation of L-Glu currents in isolated aged II SN. (A)** Representative L-Glu currents in response to perfusion of isolated aged II SN with PMA or dbcAMP, with the 5-HT-modulated L-Glu currents in aged II tail SN reproduced from Figure 1A. **(B)** L-Glu current density increased significantly following PMA (*n* = 7) and dbcAMP (*n* = 8) treatment in aged II SN; the ineffective 5-HT treatment time course is reproduced from Figure 1B. (*) denotes significant overall increase in current density at *p* ≤ 0.05 via repeated measures ANOVA. *Post hoc* comparisons responsible for these differences are summarized in results.

To test if the intracellular second messenger systems were intact or could be salvaged, we treated aged II SN with chemical analogs of second messengers downstream of G-protein initiated signaling. PMA treatment significantly increased L-Glu current density in aged II SNs (Figures 2A,B; *p* ≤ 0.05, repeated measures ANOVA; *n* = 7) to nearly the same extent as 5-HT modulated mature SN (Figure 1B), and L-Glu-evoked currents remained augmented following washout (*p* ≤ 0.05 for 5–16 min time points compared to baseline; Tukey’s *post hoc* tests). dbcAMP similarly enhanced L-Glu currents in cultured aged II SN (Figures 2A,B; *p* ≤ 0.05, repeated measures ANOVA; *n* = 8), with facilitation of L-Glu currents persisting following 10 min washout with ASW (*p* ≤ 0.05 for all time points after 8 min dbcAMP compared to baseline; Tukey’s *post hoc* tests). Whereas PMA in aged II SN resulted in 90.1% of the L-Glu-induced current facilitation caused by 5-HT treatment in mature SN, 10 min dbcAMP treatment achieved 82.3% of mature SN 5-HT-induced facilitation. Thus, activators of the PKC and PKA second messenger signaling cascades in aged II SN caused facilitation of the L-Glu currents similar to 5-HT-induced facilitation in mature SN.

### Activators of PKA and PKC Rescued Aged Tail SN Function in a Semi Intact Preparation

**Figure 3:**
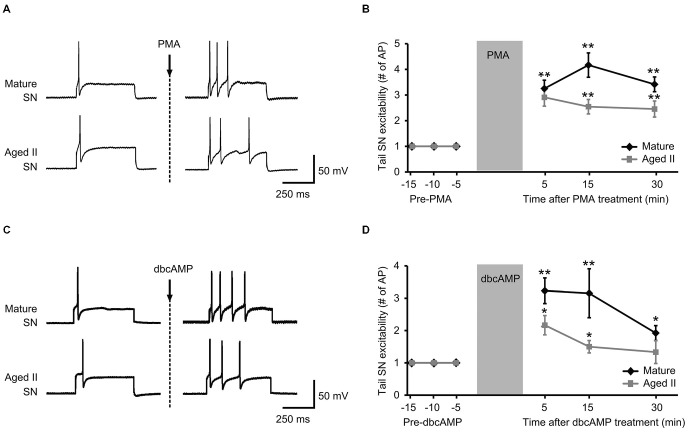
**Treatment with activators of PKA and PKC signaling rescued aged II SN performance in semi intact preparation. (A)** Representative responses in mature and aged II tail SN during depolarizing current injection before and 5 min after the end of 10 min PMA perfusion (50 nM) onto the pleural-pedal ganglion. Mature and aged II neurons were maintained at a holding potential of −43 mV during the experiment. **(B)** In both mature (*n* = 12) and aged II (*n* = 11) tail SN, the number of AP in response to depolarizing current injection was significantly increased at 5, 15, and 30 min following the end of PMA treatment. (**) denotes significant increase compared to baseline at *p* ≤ 0.01, Tukey’s *post hoc* tests. **(C)** Responses in mature and aged II tail SN before and 5 min after dbcAMP perfusion (20 μM). Mature and aged II neurons were maintained at a holding potential of −43 mV during the experiment. **(D)** In both mature (*n* = 13) and aged II (*n* = 12) tail SN, the number of AP in response to depolarizing current injection was significantly increased at 5 and 15 min following dbcAMP treatment. At 30 min after the end of dbcAMP application, excitability of mature SN was still enhanced, while aged II SN excitability had returned to baseline value. (*) and (**) denotes significant increase compared to baseline at *p* ≤ 0.05 and *p* ≤ 0.01, respectively, Tukey’s *post hoc* tests.

PMA and dbcAMP previously were shown to increase tail SN excitability and facilitate tail SN-MN transmission (Byrne et al., 1988; Kandel, 2001). We recently reported that synaptic facilitation declined during aging in the TWR circuit whether the sensitizing stimulus was electrical stimulation or 5-HT treatment (Kempsell and Fieber, 2015). The ability of isolated aged II SN to respond to PMA and dbcAMP with facilitiated L-Glu-induced currents implied that excitability modulating intracellular pathways were at least partially intact in aging, and prompted questions about whether these agents could restore TWR responses to sensitizing stimuli that were absent in aged animals. We tested PMA and dbcAMP on SN excitability in ganglia-tail preparations from mature and aged II sibling Aplysia. The PMA results are shown in Figures 3A,B. Depolarizing current of 0.3–1.5 nA induced a single AP in control conditions in mature and aged II SN. The same depolarizing pulse that produced a single AP in baseline conditions evoked a significantly greater number of AP when tested after PMA treatment in both mature (Figures 3A,B, *p* ≤ 0.01 at 5, 15, and 30 min compared to baseline, Tukey’s *post hoc* analysis; *n* = 12) and aged II tail SN (*p* ≤ 0.05 at 5, 15, and 30 min compared to baseline, Tukey’s *post hoc* analysis; *n* = 11). dbcAMP results are shown in additional mature and aged II preparations in Figures 3C,D. The same depolarizing pulse that produced a single AP in baseline conditions evoked a significantly greater number of AP when tested after dbcAMP treatment in both mature (Figures 3C,D, *p* ≤ 0.01 at 5 and 15 min, *p* ≤ 0.05 at 30 min compared to baseline, Tukey’s *post hoc* analysis; *n* = 13) and aged II tail SN (*p* ≤ 0.05 at 5 and 15 min compared to baseline, Tukey’s *post hoc* analysis; *n* = 12). Note that the dbcAMP-induced excitability increase in aged II ganglia that persisted for 15 min post-treatment returned to baseline after 30 min. Thus, activators of PKA and PKC signaling rescued aged II tail SN function, at least temporarily.

## Discussion

5-HT released at synapses during sensitization in Aplysia increases SN excitability and SN-MN transmission in tail- and gill-withdrawal reflexes via PKA- and PKC-induced signaling in both SN and MN (Brunelli et al., 1976; Walters et al., 1983b; Glanzman et al., 1989; Mackey et al., 1989; Mercer et al., 1991; Levenson et al., 1999; Marinesco and Carew, 2002; Philips et al., 2011). These elemental processes define the role of 5-HT in stimulating the formation and maintenance of memory. We have recently described changes in short-term memory for sensitization in TWR and related declines in facilitation between tail SN-MN synapses during aging, suggesting that nonassociative learning is impaired in aged Aplysia (Kempsell and Fieber, 2015). In that study, 5-HT-exposed, mature SN in intact ganglia responded to depolarizing stimuli with multiple AP compared to the single AP response before 5-HT, whereas aged SN showed no change in excitability with 5-HT. In a separate readout of SN excitability, we found here that 5-HT potentiated L-Glu-induced excitatory currents of mature but not aged isolated tail SN.

The result that 5-HT was ineffective at inducing synaptic facilitation in aged neurons suggests that the 5-HT receptor-initiated signaling cascade that increases cAMP, and results in sensitization, is compromised during aging. The compromise is somewhere upstream of cAMP, thereby almost eliminating sensitization in aged SN. An analog of cAMP that activates PKA and an analog of diacylglycerol that activates PKC imitated the effects of 5-HT on mature SN by potentiating L-Glu currents of isolated aged SN. These agents also increased the excitability of aged intact tail SN to depolarizing stimuli. Although bath application of PMA or dbcAMP most likely activated many neurons, including interneurons, the end result was enhanced tail SN excitability, even in ganglia from aged II animals. Thus stimulation of the intracellular second messenger cascade critical to learning-related facilitation in Aplysia mitigated aging SN performance declines.

While facilitation of excitation occurs via activation of intracellular signaling pathways, membrane 5-HT receptors and associated G-proteins activate these signaling cascades. Our results suggest two things about aging of facilitation in TWR. First, the second messenger cascade of specifically the SN failed in aging. Aging defects also may be present in other components of the tail withdrawal circuit, but the observation that L-Glu current potentiation of isolated aged SN by 5-HT was impaired demonstrated that the aged SN had an unmistakable defect. Second, if activators of PKA or PKC signaling were applied, permitting the intracellular second messenger cascade to proceed past the point of phosphorylation of a kinase, facilitation was observed in aged SN. L-Glu current potentiation of aged SN by activators of PKA or PKC approximated that induced by 5-HT in mature SN. The number of AP produced in response to depolarizations was restored to values approximating the facilitory effects on mature SN by activators of PKA or PKC. This suggests that a primary aging defect in facilitation of SN excitability is loss of a component upstream of PKA- and PKC-dependent signaling. It also suggests that correction at the point of what is presumed to be kinase phosphorylation is necessary to recover facilitation.

G-protein coupled signaling is altered in many animal models of aging (Stern et al., 1985; Bickford et al., 1986; Bickford-Wimer et al., 1988; Shen and Barnes, 1996; Ayyagari et al., 1998; Foster, 1999; Nicolle et al., 1999). We observed that the facilitation impairment in aged SN was repaired by components of either PKA *or* PKC dependent signaling. The defect may be altered G_s_-5-HT-receptor coupling, or reduced activation of adenylyl cyclase, leading to inadequate levels of cAMP and decreased PKA activation. The restorative effects of PMA suggested that aging in SN affects multiple second messenger signaling cascades. The PKA and PKC kinase pathways regulate several neuronal processes involving ion channels, neurotransmitter receptors, Ca^2+^ dynamics, and synaptic plasticity. Signaling components downstream of PKA and PKC may remain intact in aged tail SN.

Stimulation of second messenger signaling has been shown to reverse memory loss and physiological defects in the aging hippocampus (Bach et al., 1999). Furthermore, 5-HT receptor activation, specifically 5-HT_4_ and 5-HT_7_ receptors and the G_s_ cascade, was shown to restore age-related memory deficits in aged animals (Moser et al., 2002). Our findings support the hypothesis that deficient 5-HT receptor physiology and second messenger-dependent signaling results in age-related learning impairments and altered neuronal functioning in Aplysia.

Decreased synaptic strength during aging is the result of declines in postsynaptic responsiveness including decreases in level of expression of ionotrophic glutamate receptors (iGluR; Magnusson et al., 2002). Studies have shown a decrease in L-Glu- mediated excitatory transmission during aging corresponding to changes in the composition of subunits of major iGluR including alpha-amino-3-hydroxy-5-methylisoxazole-4-propionic acid receptors (AMPAR) and N-methyl-D-aspartate receptors (NMDAR; Barnes et al., 1992, 1997; Jouvenceau et al., 1998; Magnusson, 1998; Magnusson et al., 2000; Potier et al., 2000; Brim et al., 2013). Specifically, age-related declines in NMDAR binding density and expression of NMDAR subunits in the frontal lobe and hippocampus of rodents were associated with declines in spatial memory during aging (Magnusson et al., 2007; Zhao et al., 2009). The consequences of these changes in iGluR function during aging are decreased synaptic strength resulting in changes in cellular analogs of learning and memory including reduced induction and maintenance of long-term potentiation and lower thresholds for long-term depression (Foster, 1999; Eckles-Smith et al., 2000; Billard and Rouaud, 2007; Bodhinathan et al., 2010; Kumar and Foster, 2013; Lee et al., 2014; Guidi et al., 2015). Age-related changes in synaptic plasticity resulted in learning failure and memory loss including poor performance in spatial memory tasks (Barnes et al., 2000; Almaguer et al., 2002).

Tail SN use L-Glu as their primary excitatory neurotransmitter (Dale and Kandel, 1993; Fox and Lloyd, 1999; Levenson et al., 2000a,b; Ha et al., 2006), and the excitatory responses to L-Glu have been described (Carlson and Fieber, 2011; Carlson et al., 2012), including L-Glu-induced current amplitude declines in old tail SN (Fieber et al., 2010; Kempsell and Fieber, 2014). The latter studies suggested that losses in SN excitability are a hallmark of aging.

Although the physiological role of the L-Glu excitatory responses in tail SN is not established, the synapses of these neurons are vulnerable to synaptic facilitation and depression (Walters et al., 1983b; Stopfer and Carew, 1996), thus excitatory agonists including L-Glu may serve as neuromodulators. Subtypes of the AMPA and NMDA types of glutamate receptors as well as glutamate-mediated excitatory transmission has been shown to decrease during brain aging (Barnes et al., 1992; Jouvenceau et al., 1998; Potier et al., 2000; Clayton and Browning, 2001; Newcomer and Krystal, 2001; Barria and Malinow, 2002; Clayton et al., 2002; Magnusson et al., 2002). AMPA and NMDA receptors contribute to the induction and expression, respectively, of long-term synaptic plasticity, and age-related loss of functional receptors results in impaired synaptic plasticity and learning deficits (Adams et al., 2001; Clayton et al., 2002). As it has been previously shown that L-Glu activates NMDA-like receptors in Aplysia SN (Carlson and Fieber, 2011), it is possible that the decrements in iGluR signaling described in this study are due to NMDAR dysfunction similar to that seen in mammalian models of aging (Foster et al., 2001). Altered iGluR composition in aged Aplysia neurons likely contributes to the changes in learning and related changes in proxies for synaptic plasticity found in this study and others (Kempsell and Fieber, 2015).

## Conclusion

Neuronal proxies for synaptic plasticity including 5-HT-induced excitability and facilitation of L-Glu currents in tail SN were impaired during aging. Treatment with activators of second messenger signaling cascades rescued facilitation of L-Glu-evoked responses and increased excitability in aged tail SN, suggesting possible molecular targets with the potential to restore age-related memory impairment in Aplysia.

## Grants

This work was funded by National Institutes of Health Grant P40 OD010952, and Maytag and Korein Foundation fellowships to ATK.

## Conflict of Interest Statement

The authors declare that the research was conducted in the absence of any commercial or financial relationships that could be construed as a potential conflict of interest.

## Abbreviations

AMPAR: alpha-amino-3-hydroxy-5-methylisoxazole-4-propionic acid receptors
AP: action potential
ASW: artificial seawater
cAMP: cyclic monophosphate
dbcAMP: dibutyryl adenosine 3′,5′-cyclic monophosphate
EPSP: excitatory postsynaptic potential
iGluR: ionotrophic glutamate receptors
L-Glu: L-glutamate
LTD: long-term depression
LTP: long-term potentiation
NMDAR: N-methyl-D-aspartate receptors
PKA: protein kinase A
PKC: protein kinase C
PMA: phorbol 12-myristate 13-acetate
MN: motoneuron
PVC: pleural ventral caudal
SN: sensory neuron
TWR: tail-elicited tail withdrawal reflex
5-HT: serotonin.
